# Preoperative Controlling Nutritional Status (CONUT) score predicts short-term outcomes of patients with gastric cancer after laparoscopy-assisted radical gastrectomy

**DOI:** 10.1186/s12957-021-02132-6

**Published:** 2021-01-23

**Authors:** Yun Qian, Huaying Liu, Junhai Pan, Weihua Yu, Jiemin Lv, Jiafei Yan, Jiaqi Gao, Xianfa Wang, Xiaolong Ge, Wei Zhou

**Affiliations:** 1 Department of General Surgery, Sir Run Run Shaw Hospital, School of Medicine, Zhejiang University, 3 East Qingchun Road, Hangzhou, 310016 China; 2 Department of Medicine, GuangXi Medical College, Nanning, China

**Keywords:** Controlling Nutritional Status score, Gastric cancer, Postoperative complications, Laparoscopic surgery

## Abstract

**Background:**

An emerging prediction tool, the Controlling Nutritional Status (CONUT) score, has shown good assessment ability of postoperative outcomes in cancer patients. This study evaluated the role of the preoperative CONUT score regarding the short-term outcomes of gastric cancer (GC) after laparoscopic gastrectomy.

**Methods:**

Three hundred and nine GC patients undergoing laparoscopic gastrectomy from January 2016 to June 2019 were analysed, retrospectively. The patients were divided into two groups according to the CONUT optimal cut-off value. Clinical characteristics and postoperative complications in the two groups were analysed and evaluated. Risk factors for complications were identified by univariate and multivariate analyses.

**Results:**

A total of 309 patients underwent laparoscopic gastrectomy; 91 (29.4%) patients experienced postoperative complications. The preoperative CONUT score showed a good predictive ability for postoperative complications (area under the curve (AUC) = 0.718, Youden index = 0.343) compared with other indices, with an optimal cut-off value of 2.5. Patients with high CONUT score had a significantly higher incidence of overall complications (*P* < 0.001). Age, haemoglobin, C-reactive protein, red blood cell levels, CONUT scores, surgical procedure type, T1, T4, N0 and N3 pathological TNM classification, and pathological stages of I and III were associated with postoperative complications (*P* < 0.05). Furthermore, the preoperative CONUT score was identified as an independent risk predictor of postoperative complications (*P* = 0.012; OR = 2.433; 95% CI, 1.218-4.862) after multivariate analysis.

**Conclusions:**

The preoperative CONUT score is a practical nutritional assessment for predicting short-term outcomes in GC patients after laparoscopy-assisted gastrectomy.

## Introduction

As a major public health issue globally, gastric cancer (GC) is the third leading cause of cancer-related death [1]. Despite recent progress in the diagnosis and treatment of GC, patient prognosis remains poor. The main curative therapeutic option for GC is surgical resection [2, 3], with inevitable postoperative complications, leading to longer hospitalisation, greater expenses, poor quality of life and adjuvant chemotherapy therapy delay.

Patients with GC may have to endure unpleasant symptoms, such as early satiety, anorexia and dysphagia, caused by obstruction due to the tumour mass and chronic anaemia due to malignant ulcers. These factors result in progressive weight loss, compromised immunity and ultimately malnutrition [4]. Indeed, malnutrition is quite common and severe amongst patients with GC, especially in those with advanced GC.

Therefore, multiple nutritional assessment systems have emerged with the aim of identifying applicable parameters or tools, detecting malnutrition and predicting outcomes of patients with GC. For example, Oh et al. [5] analysed patients with GC and confirmed various perioperative nutritional parameters, including the prognostic nutritional index (PNI) and albumin (ALB), to be independent predictors of complications. Sun et al. [6] reported that ALB and neutrophils could predict postoperative overall survival (OS) in patients with GC, and Kim et al. [7] observed that the platelet-to-lymphocyte ratio (PLR) is able to predict the prognosis of GC. Other nutritional assessment tools have been reported for cancer patients, including the Nutritional Risk Screening (NRS), Skeletal Muscle Index (SMI) and Naples Prognostic Score (NPS) [8–12].

The Controlling Nutritional Status (CONUT) score was initially reported in 2005 as a useful assessment for the early detection and persistent monitoring of malnutrition [13]. The score consists of serum ALB, total lymphocyte count and cholesterol levels measurements. In recent years, several studies have shown that the CONUT score is a validated and useful assessment of nutritional status for predicting multiple cancer outcomes after surgery, including in colorectal cancer [14], hepatocellular carcinoma [15], oesophageal cancer [16], and GC [8, 17–21]. However, there was few research on the CONUT score in predicting postoperative outcomes in GC patients after radical gastrectomy. Therefore, this study aimed to assess the predictive ability of the preoperative CONUT score with regard to short-term outcomes in GC patients who underwent laparoscopic radical gastrectomy.

## Patients and methods

### Study patients

**Fig. 1:**
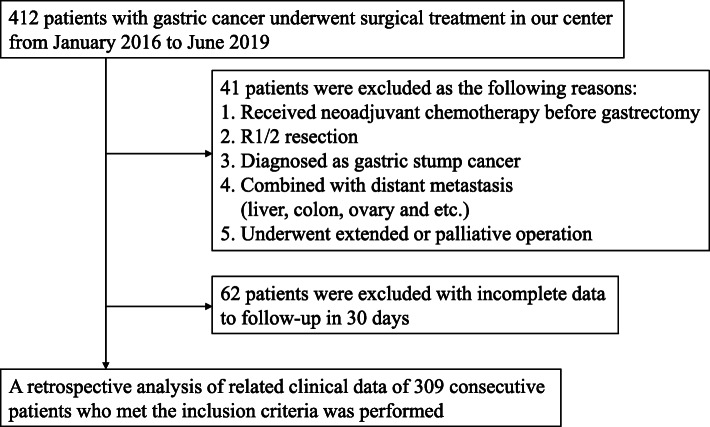
A flow chart of the inclusion process for patients with gastric cancer

Consecutive clinical records for 412 patients undergoing laparoscopic gastrectomy from January 2016 to June 2019 were initially examined in this study. The inclusion criteria were as follows: (1) gastric carcinoma confirmed by pathological diagnosis from gastroscopic biopsy, (2) curative laparoscopic gastrectomy performed and (3) age > 18 years. The exclusion criteria were as follows: (1) neoadjuvant chemotherapy before gastrectomy, (2) R1/2 resection, (3) diagnosed with gastric stump cancer, (4) GC combined with distant metastasis (liver, colon, ovary, etc.), (5) extended or palliative surgery performed and (6) incomplete data during follow-up. Ultimately, 309 patients were enrolled in the retrospective analysis. The detailed flow-chart is shown in Fig. 1. Written informed consent for the usage of clinical records was granted by each patient, as required by the Institutional Review Board at the hospital, in accordance with the ethical guidelines of the Declaration of Helsinki in 1964.

### Perioperative management

Routine case history collection, physical examination, and preoperative laboratory measurements were performed. Abdominal enhanced computed tomography and endoscopy together with tissue biopsy were carried out for the overall assessment of gastric tumours. Standard surgical laparoscopic gastrectomy with a sufficient resection margin was performed according to guidelines [3], which involved either total or distal gastrectomy coupled with systematic lymphadenectomy abiding by D level criteria. The following alimentary tract reconstruction methods were usually employed: Roux-en-Y esophagojejunostomy was performed after total gastrectomy, whereas Billroth I, Billroth II or Roux-en-Y gastrojejunostomy was selected after distal gastrectomy. For all patients, reasonable perioperative management was in line with the Enhanced Recovery After Surgery (ERAS) programme, including preoperative disease education, reducing fasting time, intraoperative use of minimally invasive techniques, fluid restriction to avoid overload, postoperative early drain removal, off-bed mobilisation and oral feeding until discharge [22–24]. Thereafter, those diagnosed with advanced gastric carcinoma were recommended to receive subsequent adjuvant chemotherapy.

### Data collection

Clinical records of baseline characteristics, laboratory data, imaging scanning examinations and pathological diagnosis were collected from a database. The CONUT score was assessed according to Table 1. PNI = 10 × serum ALB (g/dL) + 0.005 × total lymphocyte count (per mm^3^), and PLR = platelet count/total lymphocyte count. Short-term outcomes were mainly postoperative complications that occurred within 30 days after laparoscopic surgery or before hospital discharge.

**Table 1:** Assessment of malnutritional status by the CONUT score

Parameter	Malnutritional status			
	Normal	Mild	Moderate	Severe
ALB (g/dl)	≥ 3.5	3.0 ≤ ALB< 3.5	2.5 ≤ ALB< 3.0	< 2.5
Score	0	2	4	6
TLC (mg/ml)	≥ 1600	1200 ≤ TLC< 1600	800 ≤ ALB< 1200	< 800
Score	0	1	2	3
TC (mg/dl)	≥ 180	140 ≤ TC< 180	100 ≤ TC< 140	< 100
Score	0	1	2	3
Total score	0-1	2-4	5-8	9-12

*ALB* albumin; *TLC* total lymphocyte count; *TC* total cholesterol

According to the Clavien-Dindo classification system [25], mild complications included grades I and II and major complications included grades III to IV as previously described [26]. For major complications, severe active haemorrhage after surgery required emergency treatment. When persistent fever and purulent drainage occurred, an internal intra-abdominal abscess was considered. Other major intractable complications included anastomotic leakage and duodenal stump fistula. Other postoperative events included respiratory complications, cardiovascular complications and surgical site infections (SSIs) [27]. Data on cancer staging were evaluated based on the Tumour-Node-Metastasis (TNM) Classification of malignant tumour.

### Statistical analysis

Data were statistically analysed using SPSS 23.0. Quantitative variables are presented as the mean ± SD; qualitative variables are presented as numbers (percentages). Student’s *t* test or the Mann-Whitney *U* test was utilised for quantitative data. The Pearson *χ*^2^ test was applied for qualitative data. Receiver operating characteristic (ROC) curve analysis was performed to analyse the predictive ability of factors, including the CONUT score, PNI, ALB and PLR. To identify independent risk predictors for postoperative complications, factors with *P* values less than 0.05 in univariate analysis were assessed in multivariate analysis. The indicators of serum ALB, total lymphocyte count and cholesterol were excluded from multivariate analysis to avoid duplication. Significance was defined as *P* values less than 0.05.

## Results

### ROC curve of the CONUT score, PNI, ALB and PLR

**Fig. 2:**
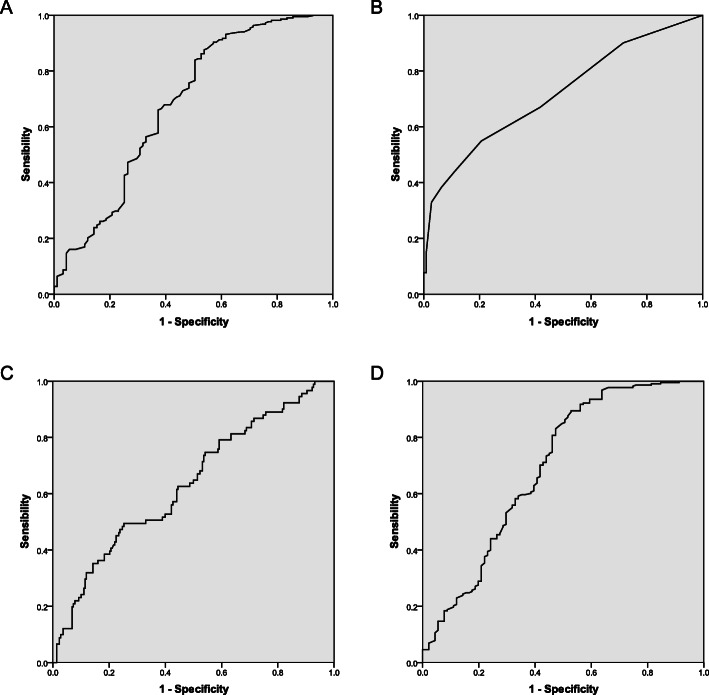
Receiver operating characteristic curve showing the capacity of ALB (**a**), CONUT score (**b**), PLR (**c**) and PNI (**d**) for predicting postoperative overall complications

In total, 309 patients were enrolled in this study. The ROC curves of the CONUT score, PNI, ALB and PLR were drawn, and the areas under the curve (AUC) were 0.718, 0.694, 0.680 and 0.635, respectively (Fig. 2). The CONUT score was the most useful predictor. The demarcated values of the CONUT score that correlated with outcomes differed from those in previous studies [8, 14–19]. In our study, the cut-off value in the prediction of postoperative complications was identified as 2.5. The Youden index of the CONUT score was 0.343, with a sensitivity of 0.549 and specificity of 0.794. The positive predictive value for postoperative complications was 52.3%; the negative predictive value was 80.8%.

### Study population and baseline characteristics based on the CONUT score cut-off value

According to the cut-off value of the CONUT score, 214 patients with scores less than 2.5 were allocated into the low CONUT score group; 95 patients with scores greater than 2.5 were allocated into the high CONUT score group. The average age of low CONUT score group patients was much lower than that of high score group patients (62.2 ± 0.7 years vs 66.2 ± 1.2 years, *P* = 0.003). The ratio of males to females in the two groups was not significantly different (155/59 vs 73/22, *P* = 0.416). Overall, body mass index (BMI) was significantly lower in patients with a CONUT score > 2.5 than in those with a CONUT score < 2.5 (21.9 ± 0.3 kg/m^2^ vs 23.1 ± 0.2 kg/m^2^, *P* < 0.001). The rate of diabetes mellitus was significantly higher in the high CONUT score group (8.9% vs 20.0%, *P* = 0.006), though no significant difference in hypertension was found (35.5% vs 43.2%, *P* = 0.201). The high CONUT score group had undergone much more previous abdominal surgery (18.7% vs 31.6%, *P* = 0.013). There were 193 (62.5%) patients who received distal gastrectomy and 116 (37.5%) who received total gastrectomy, with no significant difference between those with high and low scores (131/83 vs 62/33, *P* = 0.498).

### Comparison of clinical characteristics between patients with low and high CONUT scores

Compared to the low CONUT score group, the levels of preoperative haemoglobin (Hb) (132.5 ± 1.3 vs 104.5 ± 2.4, *P* < 0.001), ALB (40.9 ± 0.3 vs 34.6 ± 0.5, *P* < 0.001), red blood cells (RBCs) (4.38 ± 0.04 vs 3.60 ± 0.07, *P* < 0.001), platelets (PLTs) (221.4 ± 4.5 vs 206.5 ± 7.4, *P* < 0.001), total lymphocytes (1.79 ± 0.04 vs 1.04 ± 0.04, *P* < 0.001) and cholesterol (5.02 ± 0.07 vs 3.64 ± 0.08, *P* < 0.001) were lower, and the C-reactive protein (CRP) level (3.2 ± 0.6 vs 9.6 ± 1.8, *P* < 0.001) were higher in the high CONUT score group. Regarding preoperative tumour biomarkers, there were significant differences in carbohydrate antigen 125 (CA125) (*P* = 0.001) and carbohydrate antigen 199 (CA199) (*P* = 0.016) but not in carcinoembryonic antigen (CEA) (*P* = 0.769) or alpha fetoprotein (AFP) (*P* = 0.487). Patients with low CONUT scores were more likely to have pathological stage I disease (37.4% vs 23.2%, *P* = 0.014); patients with high CONUT scores were more likely to have stage III disease. The high CONUT score group experienced significantly more postoperative complications (19.2% vs 52.6%, *P* < 0.001) and had a longer postoperative stay (11.6 ± 0.5 days vs 14.1 ± 0.7 days, *P* = 0.006) than the low CONUT score group. More details are shown in Table 2.

**Table 2:** Study population and baseline characteristics of the patients sorted by the CONUT score

Characteristics	All (*N* = 309)	CONUT< 2.5 (*N* = 214)	CONUT> 2.5 (*N* = 95)	*P* value
Age, years	63.4 ± 0.6	62.2 ± 0.7	66.2 ± 1.2	0.003
Gender				0.416
Male	228 (73.8)	155 (72.4)	73 (76.8)	
Female	81 (26.2)	59 (27.6)	22 (23.2)	
BMI, kg/m^2^	22.8 ± 0.2	23.1 ± 0.2	21.9 ± 0.3	< 0.001
Comorbidities				
Diabetes mellitus	38 (12.3)	19 (8.9)	19 (20.0)	0.006
Hypertension	117 (37.9)	76 (35.5)	41 (43.2)	0.201
History of abdomen surgery	70 (22.7)	40 (18.7)	30 (31.6)	0.013
Preoperative laboratory measurements				
Hb, g/L	123.9 ± 1.4	132.5 ± 1.3	104.5 ± 2.4	< 0.001
Albumin, g/L	39.0 ± 0.3	40.9 ± 0.3	34.6 ± 0.5	< 0.001
CRP, mg/L	5.2 ± 0.7	3.2 ± 0.6	9.6 ± 1.8	< 0.001
WBC, × 10^9^/L	5.87 ± 0.09	5.99 ± 0.10	5.61 ± 0.21	0.064
RBC, × 10^12^/L	4.14 ± 0.04	4.38 ± 0.04	3.60 ± 0.07	< 0.001
Platelets, × 10^9^/L	216.8 ± 3.8	221.4 ± 4.5	206.5 ± 7.4	< 0.001
Total lymphocytes, × 10^9^/L	1.56 ± 0.04	1.79 ± 0.04	1.04 ± 0.04	< 0.001
Cholesterol, mmol/L	4.60 ± 0.06	5.02 ± 0.07	3.64 ± 0.08	< 0.001
Preoperative tumour biomarkers				
CA125, u/ml	13.6 ± 0.8	11.8 ± 0.9	17.4 ± 1.7	0.001
CA199, u/ml	27.9 ± 4.6	20.4 ± 2.8	43.9 ± 13.0	0.016
CEA, ng/ml	5.8 ± 1.6	6.1 ± 2.3	5.1 ± 1.2	0.769
AFP, μg/L	8.9 ± 5.8	11.7 ± 8.4	3.0 ± 0.5	0.487
Types of operative procedure				0.498
Distal gastrectomy	193 (62.5)	131 (61.2)	62 (65.3)	
Total gastrectomy	116 (37.5)	83 (38.8)	33 (34.7)	
Intraoperative fluid utilisation, ml	2161 ± 35.7	2209 ± 41.1	2042 ± 69.4	0.034
Operative time, min	271.8 ± 3.0	273.4 ± 3.6	268.4 ± 5.8	0.444
Estimated blood loss, ml	106.1 ± 7.3	103.0 ± 9.3	112.9 ± 11.2	0.531
T factor				
T1	91 (29.4)	78 (36.4)	13 (13.7)	< 0.001
T2	35 (11.3)	22 (10.3)	13 (13.7)	0.384
T3	42 (13.6)	23 (10.7)	19 (20.0)	0.029
T4	141 (45.6)	91 (42.5)	50 (52.6)	0.100
N factor				
N0	118 (38.2)	94 (43.9)	24 (25.3)	0.002
N1	44 (14.2)	30 (14.0)	14 (14.7)	0.868
N2	53 (17.2)	38 (17.8)	15 (15.8)	0.672
N3	94 (30.4)	52 (24.3)	42 (44.2)	< 0.001
pTNM stage				
I	102 (33.0)	80 (37.4)	22 (23.2)	0.014
II	51 (16.5)	41 (19.2)	10 (10.5)	0.059
III	148 (47.9)	88 (41.1)	60 (63.2)	< 0.001
IV	8 (2.6)	5 (2.3)	3 (3.2)	0.975
Postoperative stay, days	13.6 ± 0.5	11.6 ± 0.5	14.1 ± 0.7	0.006
Postoperative complications	91 (29.4)	41 (19.2)	50 (52.6)	< 0.001

Values in parentheses are percentages unless indicated otherwise; the other values are mean ± Sd

*BMI* body mass index; *Hb* haemoglobin; *CRP* C-reactive protein; *WBC* white blood cells; *RBC* red blood cells

### Postoperative complications in GC patients with low and high CONUT scores

The rate of postoperative complications in patients with a CONUT score < 2.5 was significantly lower than that in patients with a CONUT score > 2.5 (19.2% vs 52.6%, *P* < 0.001) (Table 3). The rate of mild complications, including sustained fever with a temperature over 38.5 °C, incision infection, persistent utilisation of total parenteral nutrition exceeding 2 weeks, postoperative blood transfusion, gastroplegia, abdominal or pelvic effusion, early postoperative bowel obstruction and urinary tract infection, was significantly higher in the high CONUT score group (8.4% vs 34.7%, *P* < 0.001). A total of 40 patients developed major complications, including postoperative active haemorrhage, intra-abdominal abscess, anastomotic leakage, duodenal stump fistula, septic shock and single organ dysfunction, and there was a significant difference between the two groups (18.7% vs 31.6%, *P* = 0.013) (Table 3). Only 1 patient died after surgery, from severe cachexia and multiple organ dysfunction syndrome (MODS). With regard to SSIs, there were 5 (1.6%) cases of surface incisional infection and 20 (6.5%) cases of deep space infection, with no significant difference between the two groups (0.9% vs 3.2%, *P* = 0.347; 5.1% vs 9.5%, *P* = 0.153).

**Table 3:** Comparison of postoperative complications in gastric cancer undergoing laparoscopic surgery with low and high CONUT score

Postoperative complications	All (*N* = 309)	CONUT< 2.5 (*N* = 214)	CONUT> 2.5 (*N* = 95)	*P* value
Overall complications	91 (29.4)	41 (19.2)	50 (52.6)	< 0.001
Mild complications (grade I to II)	42 (13.6)	17(7.9)	25 (26.3)	< 0.001
Fever> 38.5 °C after surgery	9 (2.9)	4 (1.9)	5 (5.3)	0.204
Incision infection	5 (1.6)	2 (0.9)	3 (3.2)	0.347
TPN> 2 weeks	10 (3.2)	4 (1.9)	6 (6.3)	0.091
Postoperative blood transfusion> 2 U	5 (1.6)	2 (0.9)	3 (3.2)	0.347
Gastroplegia	2 (0.6)	1 (0.5)	1 (1.1)	0.521
Early postoperative bowel obstruction	10 (3.2)	4 (1.9)	6 (6.3)	0.091
Urinary tract infection	1 (0.3)	0 (0.0)	1 (1.1)	0.307
Major complications (grade III to IV)	70 (22.7)	40 (18.7)	30 (31.6)	0.013
Postoperative active haemorrhage	16 (5.2)	10 (4.7)	6 (6.3)	0.548
Abdominal/pelvic effusion	7 (2.3)	3 (1.4)	4 (4.2)	0.264
Intra-abdominal abscess	17 (5.5)	11 (5.1)	6 (6.3)	0.676
Anastomotic leakage	9 (2.9)	5 (2.3)	4 (4.2)	0.591
Anastomotic stenosis	4 (1.3)	3 (1.4)	1 (1.1)	0.802
Duodenal stump fistula	9 (2.9)	6 (2.8)	3 (3.2)	0.864
Septic shock	3 (1.0)	0 (0.0)	3 (3.2)	0.028
Single organ dysfunction	4 (1.3)	2 (0.9)	2 (2.1)	0.768
MODS	1 (0.3)	0 (0.0)	1 (1.1)	0.307
Dead cases (grade V)	1 (0.3)	0 (0.0)	1 (1.1)	0.307
Surgical site infection, SSI	25 (8.1)	13 (6.1)	12 (12.6)	0.051
Surface incisional infection	5 (1.6)	2 (0.9)	3 (3.2)	0.347
Deep space infection	20 (6.5)	11 (5.1)	9 (9.5)	0.153
Respiratory complications	20 (6.5)	8 (3.7)	12 (12.6)	0.003
Cardiovascular complications	7 (2.3)	3 (1.4)	4 (4.2)	0.264
Postoperative stay, days	13.6 ± 0.5	11.6 ± 0.5	14.1 ± 0.7	0.006

Values in parentheses are percentages unless indicated otherwise; the other values are mean ± Sd

*TPN* total parenteral nutrition; *ICU* intensive care unit; *MODS* multiple organ dysfunction syndrome; *SSI* surgical site infection

Postoperative complications were classified from grade I to V based on the Clavien-Dindo classification system, with grade I to II defined as mild complications, grade III to IV defined as major complications

### Univariate and multivariate analyses of risk factors for short-term outcomes in GC

In univariate analysis, age, Hb, CRP, RBCs, CONUT score, type of operative procedure, pathological TNM classification of T1, T4, N0 and N3, and pathological stage of I and III were found to be risk factors with a *P* value less than 0.05. Furthermore, age (*P* = 0.037; odds ratio (OR) = 2.237; 95% confidence interval (CI), 1.048-4.774), RBCs (*P* = 0.003; OR = 0.356; 95% CI, 0.180-0.707), and CONUT scores (*P* = 0.012; OR = 2.433; 95% CI, 1.218-4.862) were identified as independent risk indicators for postoperative complications in GC after laparoscopic gastrectomy (Table 4).

**Table 4:** Univariate and multivariate analysis of risk factors associated with postoperative complications in patients with gastric cancer undergoing laparoscopic surgery

Characteristics	Postoperative complications (*N* = 91)	No postoperative complications (*N* = 218)	*P* value	Multivariate		
				OR	95% CI	*P* value
Age, year	68.2 ± 1.1	61.4 ± 0.7	< 0.001	2.237	1.048-4.774	0.037
Gender						
Male	70 (76.9)	158 (72.5)	0.418			
Female	21 (23.1)	60 (27.5)	0.418			
BMI, kg/m^2^	22.2 ± 0.3	23.0 ± 0.2	0.059			
Comorbidities						
Diabetes mellitus	14 (15.4)	24 (11.0)	0.286			
Hypertension	39 (42.9)	78 (35.8)	0.242			
History of abdomen surgery	20 (22.0)	50 (22.9)	0.855			
Preoperative laboratory measurements						
Hb, g/L	113.6 ± 2.9	128.2 ± 1.4	< 0.001	0.521	0.219-1.237	0.139
CRP, mg/L	9.0 ± 1.8	3.5 ± 0.6	< 0.001	1.193	0.500-2.849	0.691
WBC, ×10^9^/L	5.85 ± 0.19	5.88 ± 0.11	0.881			
RBC, ×10^12^/L	3.78 ± 0.08	4.29 ± 0.04	< 0.001	0.356	0.180-0.707	0.003
Platelets, ×10^9^/L	216.0 ± 7.5	217.2 ± 4.5	0.887			
CONUT score	3.7 ± 0.3	1.6 ± 0.1	< 0.001	2.433	1.218-4.862	0.012
Albumin, g/L	36.4 ± 0.6	40.1 ± 0.3	< 0.001			
Total lymphocytes, ×10^9^/L	1.33 ± 0.06	1.66 ± 0.05	< 0.001			
Cholesterol, mmol/L	4.20 ± 0.13	4.77 ± 0.07	< 0.001			
Preoperative tumour biomarkers						
CA125, u/ml	15.5 ± 1.4	12.8 ± 1.0	0.129			
CA199, u/ml	38.7 ± 13.5	23.3 ± 3.2	0.126			
CEA, ng/ml	6.2 ± 1.5	5.6 ± 2.2	0.869			
AFP, μg/L	22.5 ± 19.4	3.3 ± 0.3	0.128			
Types of operative procedure				1.345	0.740-2.444	0.331
Distal gastrectomy	49 (53.8)	144 (66.1)	0.043			
Total gastrectomy	42 (46.2)	74 (33.9)	0.043			
Intraoperative fluid utilisation, ml	2082 ± 66.6	2195 ± 42.1	0.148			
Operative time, min	272.7 ± 5.3	271.5 ± 3.7	0.853			
Estimated blood loss, ml	124.2 ± 11.8	98.5 ± 9.1	0.110			
T factor						
T1	14 (15.4)	77 (35.3)	< 0.001	1.131	0.353-3.622	0.836
T2	8 (8.8)	27 (12.4)	0.364			
T3	16 (17.6)	26 (11.9)	0.186			
T4	53 (58.2)	88 (40.4)	0.004	1.402	0.643-3.058	0.396
N factor						
N0	25 (27.5)	93 (42.7)	0.012	2.596	0.810-8.317	0.108
N1	14 (15.4)	30 (13.8)	0.710			
N2	10 (11.0)	43 (19.7)	0.063			
N3	42 (46.2)	52 (23.9)	< 0.001	1.903	0.936-3.868	0.075
pTNM stage						
I	19 (20.9)	83 (38.1)	0.003	1.141	0.302-4.311	0.846
II	10 (11.0)	41 (18.8)	0.092			
III	60 (65.9)	88 (40.4)	< 0.001	2.897	0.986-8.511	0.053
IV	2 (2.2)	6 (2.8)	0.780			
Postoperative stay, days	20.7 ± 1.4	10.2 ± 0.2	< 0.001			

Values in parentheses are percentages unless indicated otherwise; the other values are mean ± Sd

*BMI* body mass index; *Hb* haemoglobin; *CRP* C-reactive protein; *WBC* white blood cells; *RBC* red blood cells; *CONUT* Controlling Nutritional Status

## Discussion

A clinical database with a consecutive patient cohort was analysed to explain whether the preoperative CONUT score is able to effectively predict postoperative complications for GC patients after laparoscopic gastrectomy. This study found that the preoperative CONUT score is an independent risk factor for predicting postoperative complications in GC after surgery.

The prognosis of cancer is not only associated with tumour factors but also with patient status, especially nutritional status [28, 29]. The CONUT score was originally proposed by Ignacio de Ulibarri J in 2005 as an integrated scale for assessing the nutritional status of inpatients [13]. The CONUT score can reflect protein reserves, immune function and lipid metabolism. The condition of hypoalbuminaemia suggests that the body is in a stage of hypercatabolism, which is prevalent amongst cancer patients, especially those with cachexia. Lymphocytes are important cellular components of the human immune response system that help to fight tumours by inhibiting cancer cell proliferation, invasion and migration [30]. Saka et al. [31] reported that T cell exhaustion was closely associated with poor prognosis in cancer. Cholesterol plays a vital role in modulating the activity of membrane proteins, which may be associated with the occurrence and development of cancer and interactions with the body’s immune system. Additionally, Yang et al. [32] reported that cholesterol inhibited hepatocellular carcinoma invasion and metastasis by promoting CD44 localization in lipid rafts. Therefore, this assessment scale is able to provide an integrated, rapid, and low-cost nutritional evaluation of patients.

Previous studies have proposed diversified prognostic predictors for GC, such as the PNI [5, 18, 33–36] and PLR [7, 37, 38]. These nutritional score scales are based on routine parameters from blood examinations and are applied to assess the prognosis of cancer patients. In our study, we analysed the assessment capability of these scales for predicting postoperative complications with ROC curves, and the CONUT score showed the best performance. The cut-off value for CONUT in our study was 2.5, which was in line with previous studies [18–20]. For example, Hirahara et al. [19] compared the prognostic value of the CONUT score with low (≤ 2) and high (≥ 3) score with propensity score matching in patients with gastrectomy. Liu’s study involved 697 consecutive patients for stage II-III gastric cancer and concluded that the high CONUT group (≥ 3) had a significantly lower 5-year survival [20]. There was also different optimal threshold of CONUT score in other studies. For example, Ryo et al. [17] determined the cut-off value of CONUT score as 2 for predicting mortality in 2 years and overall survival. Although the optimal value was different, the CONUT score still showed a good correlation with the outcomes of GC patients [17, 39]. In addition, we identified age and RBC count as independent risk factors for complications. In other words, old age, anaemia and malnutrition had an adverse effect on short-term outcomes in patients after gastrectomy for GC, which was consistent with prior studies [39, 40].

In previous studies, most researchers have focused on long-term survival associated with the CONUT score amongst GC patients [8, 17–21], with some focus on postoperative complications. Ryo et al. [17] mentioned the incidence of some complications, such as anastomotic leakage and intra-abdominal abscess, as being related to the CONUT score. Huang et al. [39] reported that the CONUT score was a significant risk factor for total complications and 1-year survival in elderly GC patients. A meta-analysis of prognostic significance of CONUT in GC was conducted by Takagi et al., and they suggested that the preoperative CONUT score was an independent predictor of survival and postoperative complications [41]. Our results were consistent with previous studies. In our study, stratified analysis of postoperative complications was further performed to compare low and high CONUT scores. Sometimes patients suffered multiple complications. For example, after surgery, one patient suffered a sudden stomach ache and subsequent fever with abdominal tenderness and rebound tenderness as a result of duodenal stump rupture, rapidly developed a serious intra-abdominal abscess and had to undergo a second operation with suturing, irrigation and drainage. Our analysis indicated that a higher proportion of patients with a high CONUT score developed postoperative complications, especially mild complications. We speculated that patients with hypoalbuminemia, decreased lymphocytes and hypocholesterolaemia were more likely to experience negative conditions with slow tissue repair and delayed wound healing, increasing their susceptibility to infection, prolonging their reliance on parenteral nutrition support and increasing their probability of anastomotic complications and others. SSIs are infections of the incision, organ or nearby space that occur after surgery, which can be combined with complex comorbidities and antimicrobial-resistant pathogens, and increase the challenges and expenses of treatment [27]. There was no significant difference in SSIs located at the surface incision or deep space. The respiratory complications after surgery included pneumonia and hydrothorax, which occurred more frequently in the high CONUT score group, as reported by Song Ryo et al. [17]. We considered that long stays in bed and infrequent cough and sputum may be to blame. In summary, the CONUT score acts as an evaluation strategy for precise risk stratification of postoperative complications, which allows doctors to implement active nutritional interventions for GC patients.

Despite our findings, there were still some limitations of the present study. First, this single-centre study included a homogeneous cohort of patients with a fixed surgical team. Second, selection bias cannot be ruled out in a retrospective study. Finally, follow-up assessments of the CONUT score after surgery were not available, which resulted in a lack of dynamic observations of the nutrition status. Therefore, prospective multicentre studies are warranted to confirm the predictive significance of the CONUT score for GC patients compared with other commonly used nutritional assessments and to validate the effectiveness of preoperative nutritional interventions.

## Conclusion

As a simple and feasible nutritional assessment tool, the CONUT score reliably predicts postoperative complications for patients with GC after laparoscopic gastrectomy, allowing precise risk stratification and preoperative nutritional interventions before surgery.

## Data Availability

All the data can be obtained from the author by email (gxlmed@zju.edu.cn).

## Abbreviations

CONUT: Controlling Nutritional Status
GC: Gastric cancer
PNI: Prognostic Nutritional Index
ALB: Albumin
PLR: Platelet-to-lymphocyte ratio
NRS: Nutritional Risk Screening
Hb: Haemoglobin
SSI: Surgical site infection
OR: Odds ratio
TPN: Total parenteral nutrition
